# MICADo – Looking for Mutations in Targeted PacBio Cancer Data: An Alignment-Free Method

**DOI:** 10.3389/fgene.2016.00214

**Published:** 2016-12-08

**Authors:** Justine Rudewicz, Hayssam Soueidan, Raluca Uricaru, Hervé Bonnefoi, Richard Iggo, Jonas Bergh, Macha Nikolski

**Affiliations:** ^1^Centre de BioInformatique de Bordeaux, University of BordeauxBordeaux, France; ^2^Laboratoire Bordelais de Recherche en Informatique, Centre National de la Recherche Scientifique, University of BordeauxBordeaux, France; ^3^Bergonié Cancer Institute, Institut National de la Santé et de la Recherche Médicale U1218, University of BordeauxBordeaux, France; ^4^Karolinska Institute and University HospitalStockholm, Sweden

**Keywords:** targeted sequencing, third generation sequencing, patients' cohort, cancer, de Bruijn graphs, code:python

## Abstract

Targeted sequencing is commonly used in clinical application of NGS technology since it enables generation of sufficient sequencing depth in the targeted genes of interest and thus ensures the best possible downstream analysis. This notwithstanding, the accurate discovery and annotation of disease causing mutations remains a challenging problem even in such favorable context. The difficulty is particularly salient in the case of third generation sequencing technology, such as PacBio. We present MICADo, a de Bruijn graph based method, implemented in python, that makes possible to distinguish between patient specific mutations and other alterations for targeted sequencing of a cohort of patients. MICADo analyses NGS reads for each sample within the context of the data of the whole cohort in order to capture the differences between specificities of the sample with respect to the cohort. MICADo is particularly suitable for sequencing data from highly heterogeneous samples, especially when it involves high rates of non-uniform sequencing errors. It was validated on PacBio sequencing datasets from several cohorts of patients. The comparison with two widely used available tools, namely VarScan and GATK, shows that MICADo is more accurate, especially when true mutations have frequencies close to backgound noise. The source code is available at http://github.com/cbib/MICADo.

## 1. Introduction

Capturing known cancer genes by next generation sequencing, approach known as “gene panel” or targeted sequencing, is commonly used for tumor genotyping. Such studies enable the discovery of point mutations, insertions, and deletions (indels), copy number variations and gene rearrangements.

Second-generation sequencing platforms, like Illumina, 454 Life Sciences (Roche), and Life Technologies Ion Torrent (van Dijk et al., 2014), are currently widely used for diagnostic applications in cancer. Comparatively, third-generation sequencing platforms, like Pacific Biosciences (PacBio), are just now emerging in the area of biomedical research. The use of PacBio technology in clinical context has been hampered by the high rate of sequencing errors. Indeed, 17.9% error rate has been reported (Chin et al., 2011), the majority of errors being indels according to Eid et al. (2009). However, this high error rate is mitigated by the use of single-molecule circular sequencing, resulting in error-corrected consensus sequences, namely ccs reads (Jiao et al., 2013). This approach reduces error rate up to 2.5% in raw ccs reads. Another reported positive characteristic of PacBio technology is the absence of context-specific errors that affect other short-read sequencing platforms (Carneiro et al., 2012) and potentially generate false positive variation calls. However, in the case of targeted sequencing, the polymerase chain reaction (PCR) step induces context-specific errors (Robasky et al., 2014) thus potentially reducing the benefit of stochastic nature of PacBio sequencing errors. Indeed, PCR process can prevent distinction between real mutations and context-specific polymerase artifacts, this phenomenon being increased by low input and/or poor quality DNA (Peng et al., 2015). This biais becomes particulary salient during bioinformatics processing if the analysis algorithms assume that errors occur randomly (Schirmer et al., 2015).

The goal of variant calling is to identify positions where at least one of the bases differs from the reference genome, the main types of such short differences being Single Nucleotide Variations (SNVs) and indels. SNPs are SNVs that are present in more than 1% of the population and most often do not have any impact on health. In the context of cancer data, a somatic point mutation – called somatic SNV – is an alteration which occurs in a tumor sample, the control allele being the wild type. Usually, somatic SNVs are identified by comparing a tumor sample with its corresponding normal sample. A large number of tools for variant calling has been developed in recent years (Leggett and MacLean, 2014; Pabinger et al., 2014), including methods specific for the identification of somatic mutations in cancer (see Gonzalez-Perez et al., 2013; Wang et al., 2013 for review). A number of comparative studies have shown that variant callers available within the GATK pipeline (McKenna et al., 2010) show the best performance (see e.g., Pirooznia et al., 2014; Yi et al., 2014). For cancer data, VarScan (Koboldt et al., 2012) is a popular choice. A recent study (Warden et al., 2014) has compared the performance of these two tools and has shown a significant overlap of high-quality GATK and VarScan variant calls.

Despite the existence of these numerous computational solutions, calling somatic mutations in cancer data remains challenging due to a number of factors like technical artifacts, sequencing errors, biases of alignment algorithms, DNA contamination (control samples contaminated with tumor DNA), and tumor heterogeneity. This issue is even more salient for the third generation sequencing data, such as PacBio. Indeed, since very high read depths are required for achieving sequence accuracy close to that of Illumina and Ion Torrent (Quail et al., 2012), variant calling potentially suffers from high false positive and negative rates. Moreover, variant callers are notorious for encountering difficulties with indels (Zook et al., 2014), which is the most common error type in PacBio sequencing data. These issues can be circumvented through the use of *de Bruijn graphs* (DBG) structure. Indeed, some new approaches based on the DBG structure have recently emerged (Iqbal et al., 2012; Rizk et al., 2014; Uricaru et al., 2015) but either remain specific to the detection of one variation type or are not appropriate for cancer data. Therefore, only few variant callers are recommended for PacBio data, such as the aforementioned GATK pipeline and the PacBio's Quiver algorithm.

In the clinical setting, distinguishing between true mutations and artifacts introduced by sequencing errors and variant calling tools, is essential. Indeed, systematic molecular characterization of relevant genes within patient cohorts is key for understanding cancer initiation and progression. When sequencing data obtained from a patients' cohort is analyzed for the presence of SNVs, identification of genetic variants is performed for each patient's sample individually. These individual results can be further jointly analyzed in the downstream statistical analysis. Such standard analysis workflows do not fully exploit the whole tumor cohort raw data. Concomitantly, multiple studies have shown the existence of specific error profiles in second generation sequencing. For example, GC rich regions and the extremities of sequence reads exhibit higher error rates (Allhoff et al., 2013), and both Roche 454 and Ion Torrent encounter difficulties in the homopolymer regions (Ross et al., 2013). Based on these observations, Bansal (2010) has proposed a statistical method that identifies rare and common variants in DNA pools of diploid individuals. However, the diploidy requirement prevents its application to cancer data where samples are highly heterogenous.

To overcome such difficulties in PacBio clinical applications, some studies develop *ad-hoc* solutions to perform their analyses. For example, in order to take advantage of the long read technology for the analysis of highly polymorphic regions such as e.g., human leukocyte antigen (HLA) genes, the authors must resort to extremely stringent filtering of sequencing reads thus avoiding noisy data, but also potentially getting rid of useful information (Shukla et al., 2015). When classical variant calling is used, such as GATK, the authors still make use of additional *ad-hoc* strategies. In Orkunoglu-Suer et al. (2015) the authors completely exclude indels known to be a particular difficulty of PacBio sequencing, while Smith et al. (2012) restrain the analysis to certain loci. There is thus a lack of a generic and efficient algorithmic solutions for this application niche.

In this manuscript we pursue the idea that analyzing sequencing data produced for a cohort of patients as a whole should make it possible to distinguish between real patient mutations and other alterations, including sequencing errors. Indeed, one sample can be analyzed in the context of the whole dataset, serving itself as statistical basis for filtering out systematic alterations. This idea is even more pertinent in the case of sequencing data carrying non-uniform sequencing errors as, shown in this paper, it is the case for PCR targeted PacBio data. We introduce a method called *MICADo* that distinguishes patient-specific from cohort-specific alterations and show that it efficiently performs even on samples carrying SNVs in the context of contamination by germline DNA and in the presence of technologic artifacts. MICADo is based on the well-known representation of NGS sequencing reads, DBG, which provides the double advantage of circumventing the alignment step required by most SNV callers and avoiding additional biases due to the alignment itself. In theory, MICADo can be used on any kind of sequencing data, however its advantage with respect to other variant calling methods should be lesser in the case of short read datasets with low error ratios. Therefore, MICADo was evaluated on PacBio sequencing datasets: (i) a novel sequencing of *TP53* of a breast cancer cohort, (ii) a publicly available dataset of *FLT3* sequencing of an acute myeloid leukemia cohort, and (iii) a synthetic dataset.

## 2. Materials and methods

### 2.1. MICADo approach

Our approach, called MICADo, takes as input the reference sequences for a gene of interest and several read sets corresponding to the gene panel sequencing of a cohort of patients. This method allows for accurate detection of patient mutations in such targeted sequencing data and their distinction from other alterations. MICADo is built of three major steps.

First, the reference and the cohort sequencing data are efficiently represented with de Bruijn graphs. A DBG, widely used in NGS data processing methods (Pevzner et al., 2001; Compeau et al., 2011) is a directed graph that depicts the redundancy encountered in a read set by representing all *k* length words contained in the data, i.e., *k*-mers, as vertices in the graph, and all *k* − 1 overlaps between *k*-mers as directed edges in the graph. Here we use an extension of the classical DBG, meaning a *colored* DBG (Iqbal et al., 2012). A colored DBG comprises several read sets and has multi-colored vertices meant to keep track of the datasets to which every *k*-mer belongs. We build a colored DBG *reference graph* for the gene reference sequences, including splicing variants and known SNPs. The vertices of this graph are colored according to their original reference sequences. We then build a colored DBG *sample graph* per patient dataset. The vertices of the sample graph are colored according to the patient dataset, thus with only one color.

**Figure 1:**
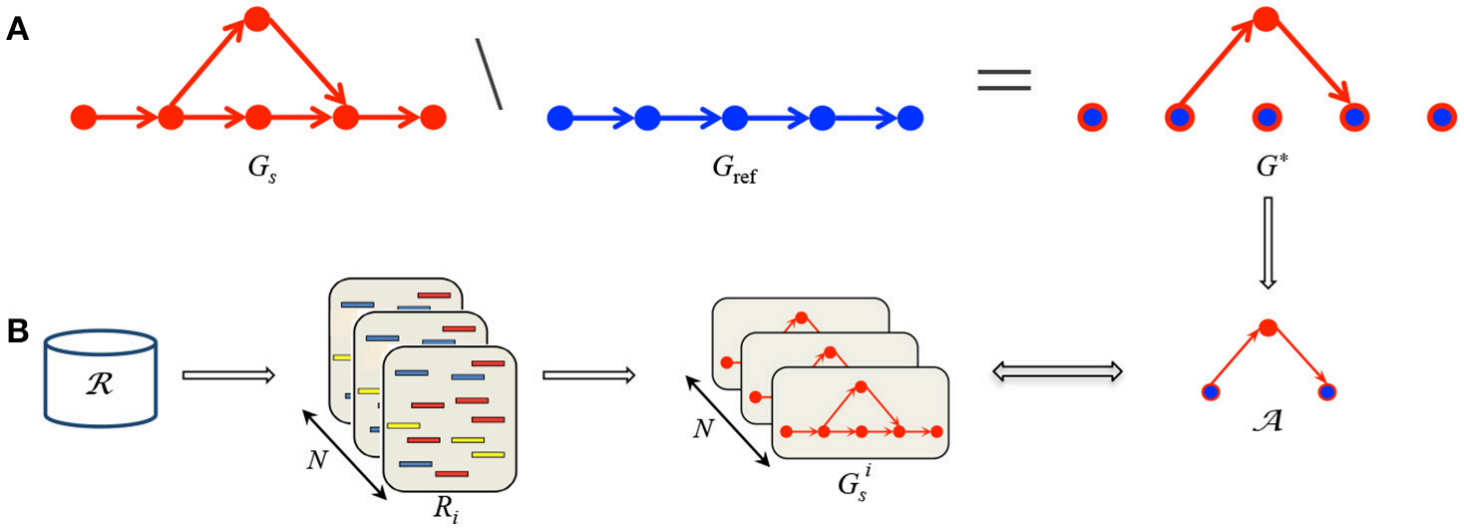
**Summary of MICADo. (A)** *G*_ref_ and *G*_*s*_ are respectively constructed from multiple reference sequences and the sample read set. **(B)** After capturing differences between *G*_*s*_ and *G*_ref_ in G^*^, the goal is to decide whether they are sample specific by checking their presence in random samples *R*_*i*_ constructed from the whole readset of the cohort R.

Second, we compare the reference graph to each sample graph in order to highlight paths in the sample graph that are not present in the reference graph, i.e., alternative paths, see Figure 1A.

Finally, for each alternative path, we verify whether it is specific to the sample or common within the cohort. This is achieved by generating random samples populated by reads from the whole cohort and checking the presence of each alternative path previously identified within these random sets, see Figure 1B. MICADo is implemented as a python program (see Supplementary Section 1 for more details).

### 2.2. Datasets

MICADo was tested on three datasets: *TP53* sequencing PacBio dataset, *FLT3* PacBio sequencing dataset, and a synthetic dataset.

#### 2.2.1. *TP53* sequencing data

The p53 protein is encoded by the tumor suppressor gene *TP53*, the most frequently mutated gene in human cancers (Olivier et al., 2010), given its major implication in response to cellular stress. Most of mutations are found in its DNA Binding Domain used to activate genes involved in apoptosis, growth arrest, or senescence of highly damaged cells (Brosh and Rotter, 2009).

p53 sequencing data is a secondary output of the original EORTC 10994/BIG 1-00 study, registered under NCT00017095 https://clinicaltrials.gov/ct2/show/study/NCT00017095. For the original study and primary data, (see Bonnefoi et al., 2011; Iggo et al., 2013).

We have sequenced 48 samples of *TP53* transcripts extracted from breast tumor biopsies (Bonnefoi et al., 2011) by PacBio circular sequencing (PacBio RSII; P4-C2 chemistry). The PacBio sequencing data is available from the NCBI SRA database under the accession number SRP064161 BioProject PRJNA290142. The main interest of this dataset for testing the accuracy of SNV detection is the existence of SNV calling results obtained from 454 Roche sequencing data generated for the 48 samples (available from NCBI SRA database under the accession number SRP020456, BioProject PRJNA193388; see Iggo et al., 2013 for details). Moreover, for these 48 samples there exists a classification into three categories (based on the percentage of red colonies in the yeast assay, (see Bonnefoi et al., 2011; Iggo et al., 2013). These categories are:

- *negative control*: 12 samples considered to be wild type;
- *positive control*: 18 samples that are mutated with high rate of altered reads;
- *difficult group*: 18 samples that are mutated with low rate or complex mutations.

Sequencing was centered on the DNA Binding Domain of the protein. *TP53* transcripts were split into two distinct fragments, 482 nucleotides long N fragment and 454 nucleotides long C fragment. After demultiplexing and primer cutting (see Supplementary Section 2 for more details), the number of reads varies from 132 to 8372 for the C fragment and from 89 to 8771 for the N fragment with respective averages of 974 and 826.

#### 2.2.2. *FLT3* sequencing data

The receptor tyrosine kinase *FLT3* is highly involved in the development of stem cells and in the immune system (Gilliland and Griffin, 2002). Mutations of *FLT3* have been detected in about 20% of patients with acute myeloid leukemia (AML) and are associated with poor prognosis (Smith et al., 2012).

This dataset is composed of 19 samples obtained by PacBio circular sequencing of *FLT3* transcripts for 8 AML patients before treatment and the same patients after relapse, and 3 healthy individuals with no cancer history. The data is available from NCBI SRA database under the accession number SRP011010 Bioproject PRJNA85103. All patients present an internal tandem duplication (ITD) which are co-located but patient specific and associated after relapse with new point mutations; a complete description of mutations carried by each patient can be found in Smith et al. (2012). Sequencing was done on the *FLT3* kinase domain which is 1346 nucleotides long, with an average of 1348 reads per sample (the number of reads varies from 59 to 4856 reads).

#### 2.2.3. Synthetic data

Synthetic samples with artificially inserted alterations were generated from the pooled *TP53* dataset as follows. Approximately 20*k* reads from all the *TP53* samples from the negative control group were first pooled in a single file and mapped with GMAP against the *TP53* transcript variant 1 (NCBI Reference Sequence: NM_000546.5). To build a synthetic sample, we first sample *n* reads (*n* ranging from 150 to 1000) from all the mapped reads (chosen with uniform probability), we then sample *i* positions (*i* ranging from 1 to 3) to alter between the minimal and the maximal mapped positions. For each of the *i* alterations, we choose with uniform probabilities between a mismatch, an insertion and a deletion, as well as an alteration length *l* (*l* is 1 for mismatches and ranges from 1 to 5 for insertions or deletions). In the case of insertions, we randomly generate a *l*-nt sequence. Finally, we generate a fastq file with *n* reads, out of which a fraction φ (φ ranging from 3 to 80%) harbors the same randomly generated alterations. The base qualitiy of each alteration was fixed to the highest base quality.

### 2.3. Construction of de bruijn graphs

A DBG is a directed graph whose vertices are words of a given length *k*, called *k*-mers, and whose edges connect words overlapping by *k* − 1 letters. Two definitions for *de Brujn* graphs are encountered in the literature, depending on the way edges are dealt with: the implicit definition that infers the edges from the vertices, i.e., every *k* − 1 overlap is accounted for whether the corresponding *k*-mers are consecutive or not within the reads (Uricaru et al., 2015), and the explicit formulation that specifically represents the edges in addition to the vertices, in which case only *k* − 1 overlaps between consecutive *k*-mers present in the reads can be considered (Iqbal et al., 2012). In this work we employ the explicit formulation. Also, it is commonly accepted that a vertex in a DBG represents both the *k*-mer and its reverse complement. Here, as reverse reads are reverse complemented prior to building the graph (see Supplementary Section 2), we solely represent *k*-mers (and not their reverse complements).

Moreover, we use an extended version of the DBG, a *colored* DBG (Iqbal et al., 2012), best suited to the multi-sample case. A colored DBG takes several read sets corresponding to multiple samples, and aggregates them in a union DBG, while coloring the vertices with respect to the samples they belong to. A more formal definition follows below.

DEFINITION 1. *For n given sets of sequences S*_1_*, …, S_n_ and k* ≥ 2*, the corresponding colored de Bruijn graph G* = 〈*V, E, l*〉 *is such that its set of vertices V is composed of all k-mers of S*_1_*, …, S_n_, its edge set is*

$$\begin{array}{l} E=\{(v,w):v,w\in V\;\text{and}\;v_{2}\ldots v_{k}=w_{1}\ldots w_{k-1}\;\text{and}\\ \;\;\exists i\in [1,n]\text{s}.\text{t}.\exists s\in S_{i},v_{1}v_{2}\ldots v_{k}w_{k}\subseteq s\}, \end{array}$$

*and the labeling function* l:V→P(L) *provides colors for the vertices with L being the set of n labels (colors) corresponding to the n sets of sequences.*

When it is clear from the context, we will use a shortcut notation *v* = *w* for the equality of *k*-mers that are encoded by the corresponding vertices and omit the notation of the labeling function *l*.

To sum up, in this work we employ the definition of DBG that (i) is extended by a labeling function *l* to distinguish between reference sequences (such as SNPs and splicing variants) and (ii) explicitely encodes edges corresponding to *k*-mers present in the sequences. that are truly present in the sequences. Basically this implies that every walk in the graph corresponds to a sequence that exists in at least one dataset and its labels identify the datasets to which the sequence belongs to.

For a gene of interest, its *reference graph*, noted *G*_ref_ = 〈*V*_ref_, *E*_ref_, *l*〉, is a *n*-colored DBG constructed from the *k*-mer decomposition of the *n* sequences corresponding to its splicing variants and known SNPs. A *sample graph*, noted *G*_*s*_ = 〈*V*_*s*_, *E*_*s*_〉, is a 1-colored DBG, thus a simple DBG, constructed from the sequencing reads of one sample. For the sample graph we define the *read support* of a vertex *v*, denoted as *r*(*v*), as the number of reads the corresponding *k*-mer appears in. In order to discard indisputable sequencing errors, vertices having a read support below a fixed threshold are removed from *G*_*s*_ (see Supplementary Section 7).

### 2.4. Alternative path search

**Figure 2:**
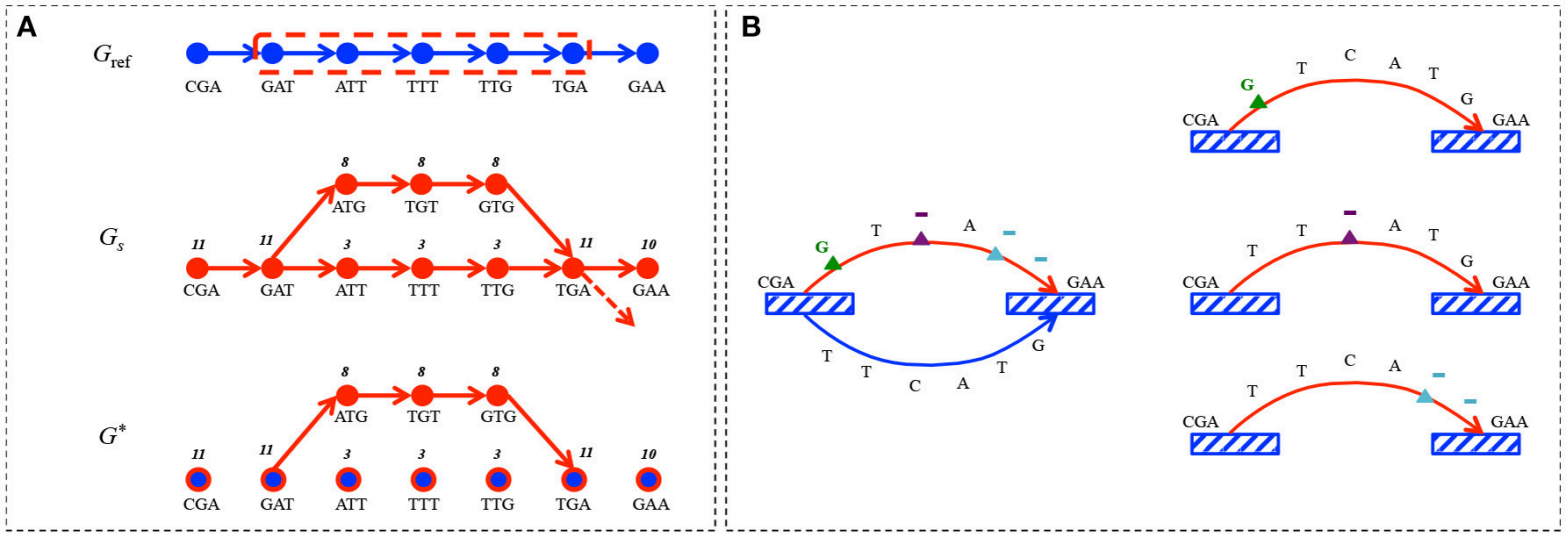
**Alternative path search**. **(A)** *G*_ref_ encodes the sequence CGATTTGAA for *k* = 3. The sequencing set corresponding to the sample is composed of 8 reads containing CGATGTGAAsequence, 2 reads with CGATTTGAA and 1 read containing CGATTTGA sequence followed by something else than A; *G*_*s*_ represents this read set for *k* = 3. The difference graph G^*^ contains the alternative path *p*_*a*_ encoding the subsequence ATGTG with its read support *r*(*p*_*a*_) = 8, as well as the singleton vertices (blue dots circled in red) for shared *k*-mers between *G*_ref_ and *G*_*s*_, but with no alternative path between them. The reference path corresponding to *p*_*r*_ is circled in red in *G*_ref_. **(B)** An alternative path *p*_*a*_ encoding the sequence CGAGTAGAA is identified with the corresponding *p*_*r*_ encoding CGATTCATGGAA. *k*-mers common to *p*_*a*_ and *p*_*r*_ are depicted by striped boxes. *p*_*a*_ carries 3 alterations, corresponding to the following edit operations: δ_1_ - substitution of T by a G (green triangle), δ_2_ - deletion of C(lilac triangle) and δ_3_ - deletion of TG(blue triangle). *p*_*a*_ is thus split in three atomic alternative paths, each carrying only one alteration.

Given the reference sequences and the sample reads modeled by *G*_ref_ and *G*_*s*_, detecting sequence alterations (insertions, deletions and mismatches) comes to capturing the differences between these graphs. This means that we have to identify paths, i.e., sequences of vertices, present in *G*_*s*_ but absent in *G*_ref_, that we call *alternative paths*. Basically, an alternative path corresponds to a sequence of *k*-mers (vertices) within the sample that differ from the reference sequence except for the two anchoring *k*-mers which are common to both the sample and the reference (see Figure 2A).

DEFINITION 2. *We define the* difference graph G^*^ = 〈V^*^, E^*^〉 *to be such that* $V^{*}=V_{s}\cup V_{ref}$ *and* $E^{*}=E_{s}\backslash E_{ref}$*. A path* $p_{a}=\left(v_{1}^{*}\ldots v_{n}^{*}\right)$ in G^*^ *is said to be an alternative path if there exists a path p*_*r*_ = (*v*_1_ … *v*_*m*_) *in G*_ref_ *such that* $v_{1}^{*}=v_{1}$ *and* $v_{n}^{*}=v_{m}$*.*

We generalize the notion of *read support*, originally defined on vertices, to paths in the sample graph: the *read support* of a path *p* is the number *r*(*p*) of reads from the sample in which the corresponding succession of *k*-mers composing *p* appears. We say that a read supports a path if and only if it supports all *k*-mers composing the path.

An alternative path *p*_*a*_ can carry *n* ≥ 1 alterations with respect to its reference path *p*_*r*_. We use the Levenshtein distance lev(*p*_*a*_, *p*_*r*_) between the sequences defined by *p*_*a*_ and *p*_*r*_, to determine the minimal set δ_*i*_ (with 1 ≤ *i* ≤ *n*) of edit operations (deletions, insertions and substitutions) that transform *p*_*a*_ into *p*_*r*_. This defines the set of *atomic alternative paths* corresponding to {δ_*i*_(*p*_*a*_)} (see Figure 2B). The read support of these atomic alternative paths is set to be equal to *r*(*p*_*a*_).

In the following, the set of tuples corresponding to the alterations computed for a given sample is denoted by $A=\left\{\langle p_{a},p_{r},c\rangle \right\}$, where *p*_*a*_ is an atomic alternative path in G^*^, *p*_*r*_ its corresponding reference path in *G*_ref_, and *c*(*p*_*a*_) its *count ratio* with *c*(*p*_*a*_) = *r*(*p*_*a*_)/(*r*(*p*_*a*_) + *r*(*p*_*r*_)), where *r*(*p*_*r*_) is computed in the sample graph. For example, in Figure 2A the count ratio for the alternative path *p*_*a*_ is computed as *c*(*p*_*a*_) = 8/(8 + 3) = 0.72. Notice that *p*_*a*_ is atomic as it carries only one alteration (G vs. T mismatch).

Moreover, an alternative path has to be *simple*, that is composed by at most two vertices from *V*_ref_, meaning that composite paths are not considered. The existence of multiple reference sequences can engender a combinatorial explosion of reference paths for a given alternative path. Consequently, for *p*_*r*_ we retain the reference path that maximizes the intersection, in terms of supporting reads, between the alternative path and the reference paths.

To construct A, we apply the alternative path search algorithm (Algorithm 1) presented below.

**Algorithm 1:** Alternative path search

**1** A = ∅
**2** *V*_start_ = {*v* ∈ V^*^ | deg^+^(*v*) > 0} // start vertices
**3** *V*_end_ = {*v* ∈ V^*^ | deg^−^(*v*) > 0} // end vertices
**4 for each** *v_s_* in *V*_start_ **do**
**5** **for each** *v_e_* in *V*_end_ **do**
**6** compute *A*, the set of alternative paths *p_a_* between *v_s_* and *v_e_* in G^*^
**7** **if** *A* ≠ ∅ **then**
**8** retrieve the best reference path *p_r_* between *v_s_* and *v_e_* in *G*_ref_
**9** **for each** *p_a_* ∈ *A* **do**
**10** compute *A*′ = {δ_*i*_(*p_a_*) | *p_a_* ∈ *A*} the set of atomic alternative paths
**11** **for each** $p_{a}^{\prime }$ ∈ *A*′ **do**
**12** *c* = *r*($p_{a}^{\prime }$)/(*r*($p_{a}^{\prime }$) + *r*(*p_r_*))
**13** A = A ∪ 〈$p_{a}^{\prime }$, *p_r_, *c*〉*
**14 return** A

**Figure 3:**
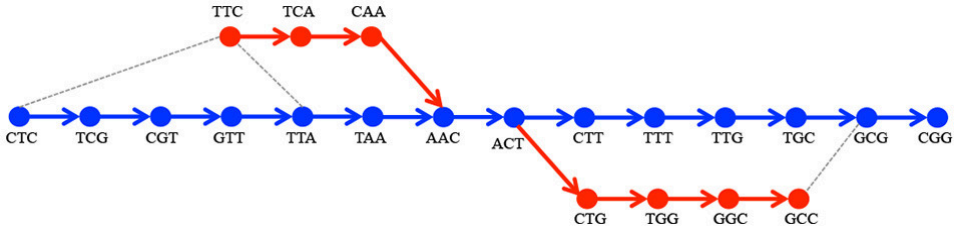
**Processing of tips**. Given the *G*_ref_ encoding the sequence CTCGTTAACTTTGCGG(in blue) and *G*_*s*_ encoding TTCAACTGGCC(in red), there are two *tips*: TTC and GCC. Two alternative paths are $p_{a}^{1}$ corresponding to TTCAAC and $p_{1}^{2}$ corresponding to ACTGGCC. For TTC the closest *k*-mers among those on the path from CTC to AAC in terms of Levenshtein distance are CTC and TTA(depicted by gray dotted lines). We choose TTA since the paths |CTC…AAC| = 6 and |TTA…AAC| = 2 - the latter path being the closest in length to $p_{a}^{1}$. In the same manner we choose GCG as anchor for GCC.

Notice that certain *v* ∈ *V*_start_ may not belong to *V*_ref_. We call such start or end vertices *tips*. Tips may appear in G^*^ for three reasons: (i) the presence of reads that do not start or end at the beginning, respectively at the end of the region of interest, (ii) the presence of reads that carry alterations at the very beginning or end of their sequence, and (iii) because of the removal from *G*_*s*_ of vertices having a read support below a fixed threshold. For the algorithm to work in all cases, we have to define an appropriate reference path for a *p*_*a*_ starting or ending with a tip. This is done by a heuristic lookup for the most plausible reference path as shown in Figure 3. To do this, we look for *anchors* of these tips in *G*_ref_. These anchors are defined as vertices that belong to paths between the start node in *G*_ref_ and the corresponding *v*_*e*_ (symmetrically, *v*_*s*_) having the smallest Levenshtein distance between the associated *k*-mers. If there is more than one such vertex, we choose the one defining the *p*_*r*_ that is the closest in length to *p*_*a*_.

### 2.5. Alternative path specificity and variant calling

Once the set of atomic alternative paths A is computed, MICADo partitions it into sample-specific alterations (i.e., mutations) and other paths corresponding either to non-specific alterations (recurrent within the cohort) or to sequencing errors. To this end, we use a permutation test based on rearranging reads and sample assignments. This approach is justified by the following two assumptions.

1. Mutations are assumed to be independent across samples and non-recurrent across the cohort. This is particularly true for loss of function mutations (such as those leading to the inactivation of *TP53*).
2. Sequencing errors are supposed to be distributed non-uniformly and are recurrent across samples. This is known to be true for the base call errors within homopolymers by second generation sequencing targeted strategies and we suppose it to hold for PacBio targeted data.

Based on these assumptions, we can determine whether an atomic alternative path *p*_*a*_ is specifically associated with a given sample by capitalizing on the background information contained in the cohort. More specifically, an atomic alternative path *p*_*a*_ is specific to a sample graph *G*_*s*_ if it is rare in the whole set of reads for the cohort, denoted R. Mutations are Levenshtein edit operations between sample-specific atomic alternative paths and the corresponding reference paths.

We measure the probability of this association between *p*_*a*_ and *G*_*s*_ by computing the count ratio *c*(*p*_*a*_) statistics under the null hypothesis of no association between reads and samples. For an observed *c*(*p*_*a*_), we generate *N* random samples *R*_*i*_ by sampling without replacement reads from R. For each of the *R*_*i*_, we compute the corresponding ratio *c*_*i*_(*p*_*a*_), that is the count ratio of *p*_*a*_ observed in the graph $G_{s}^{i}$ that encodes *R*_*i*_. We iterate this step to obtain a distribution of *c*_*i*_(*p*_*a*_) under the null hypothesis, and therefore deduce a *p*-value by comparing this distribution and the sample read counts for each alteration. Once we have performed *N* resamplings, we can reject the null hypothesis of no association between the sample and the path if less than α-percent of all *c*_*i*_(*p*_*a*_) are smaller than *c*(*p*_*a*_), where α is the required significance level (0.01 in our experiments). In such cases, we identify the altered nucleotides by identifying edit operations (insertions, replacements or deletions) between *p*_*r*_ and *p*_*a*_ and the associated *p-value* $p=\frac{\left|\left\{c_{i}(p_{a})>c(p_{a})\right\}\right|}{N}$ for this alteration. Finally, following the observation that most *c*_*i*_(*p*_*a*_) were normally distributed during our simulations and executions, we also determine a – standardized – z-score for *c*(*p*_*a*_) and only consider alterations for which the ratio *c*(*p*_*a*_) is at least 10 standard deviation away from the mean *z*(*c*(*p*_*a*_)) ≥ 10.

## 3. Results

Three pipelines based on GATK, VarScan, and MICADo (see Supplementary Sections 3 and 5 for details on how these pipelines were executed) were evaluated on both synthetic and real data. Even if Quiver algorithm is specifically developed for PacBio data, it was not suitable for our evaluation since it assumes a haploid sample with no admixture (see PacBio FAQ pages).

### 3.1. Evaluation on synthetic data

We evaluated the ability of the three pipelines to recover artificially inserted somatic mutations. We developed to this end a simple reads simulator based on sampling reads from the *TP53* dataset. Although, numerous synthetic read simulators for NGS data are available, we opted to use a simple non-parametric sampling-altering scheme based on existing samples in order to preserve as much as possible sequencing biases present within the data. We generated 9245 synthetic datasets by sampling and altering reads from the pool of negative control samples belonging to the *TP53* dataset (see Section 2.2.3 and Supplementary Section 6 for details). Moreover, any identified variants corresponding to known SNPs (described in Supplementary Section 2) were excluded from this analysis.

**Figure 4:**
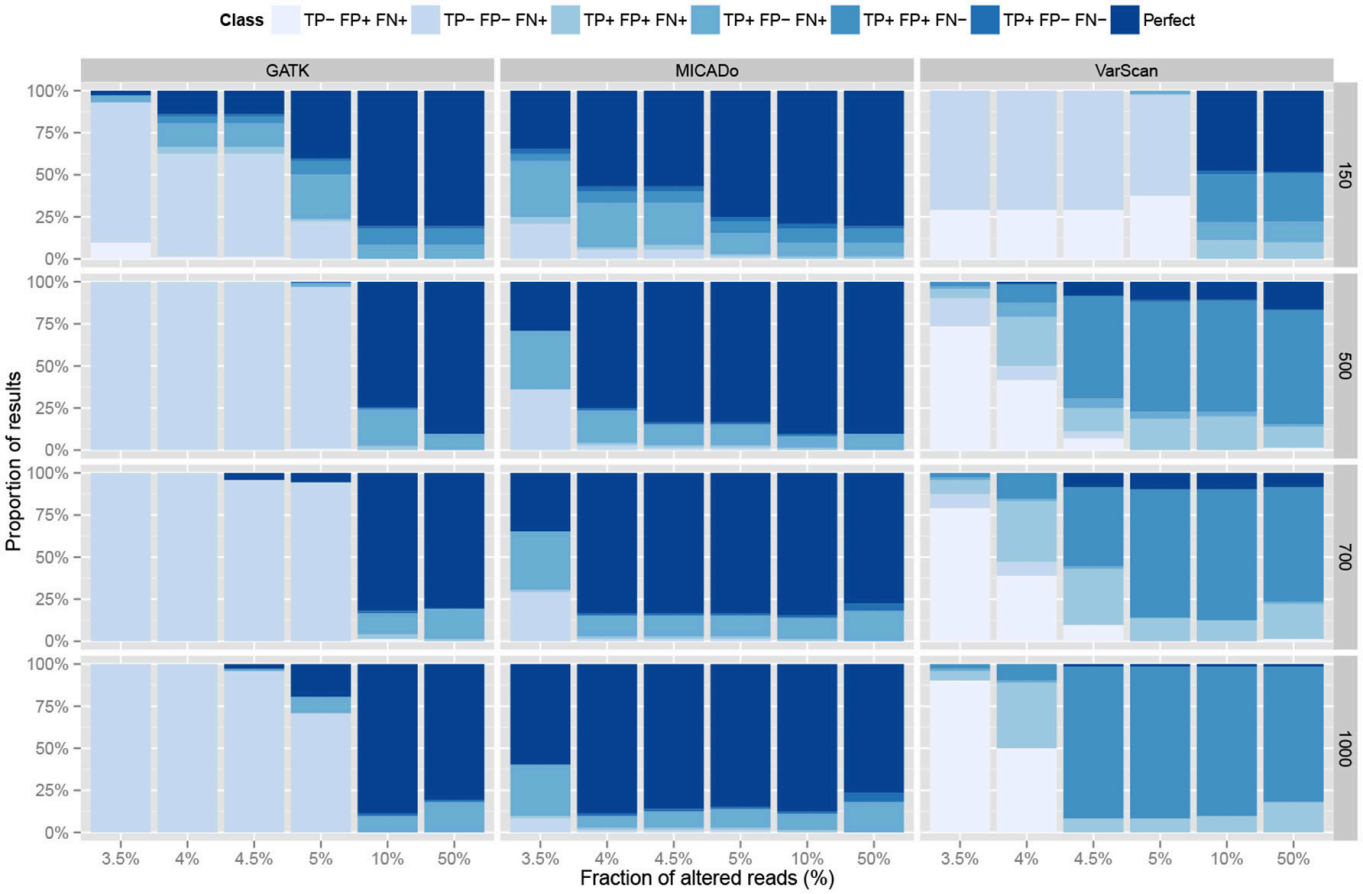
**Comparison of three pipelines on synthetic data**. Three pipelines, GATK, MICADo, and VarScan (vertical panels) were run on synthetic data containing from 1 to 3 inserted alterations, harbored by a varying fraction of altered reads (x-axis) among a total of 150, 500, 700, and 1000 reads (horizontal panels). TP- label indicates no True Positives and TP+ indicates that at least one True Positive was identified. The same notation applies for False Positives (FP) and False Negatives (FN). We identify the results where all the true positives were retrieved, and only them, as “Perfect.” Each result thus belongs to one out of 7 classes (sequential colors) and we report the proportion of results for each class (y-axis).

The three pipelines were evaluated by computing, for each sample, the number of true positives (number of correctly identified mutations), false positives (number of wrongly identified mutations), and false negatives (number of missed mutations). The results are reported in Figure 4. In terms of precision, GATK and MICADo offer similar performances when more than 10% of the reads are altered. VarScan on the other hand, systematically generates false positives (from 1 to 8 per sample), which is not the case for GATK and MICADo.

When samples contain a limited fraction of altered reads (<5%), we observe that the vast majority of mutations are not identified by either GATK or VarScan (the first four classes with “FN+” label depicted in Figure 4 indicate that at least one mutation was missed). For VarScan, this is expected, since we set the threshold of the minimum variant allele frequency to 5%; if not, the results are riddled with false positives. On the contrary, MICADo was run with a lower threshold of 3% minimum variant allele frequency and still adequately identifies both with high precision and recall the majority of mutations even when as few as 3.5% of the total number of reads contain these mutations.

Note that synthetic samples were generated by sampling from the negative control group of the *TP53* dataset that were qualified as being “wild-type” by an independent sequencing run as well as by a yeast colony assay test (see Section 2.2 for details). Therefore, any variant found in the negative control group is either a known SNP or is considered to be a false positive. Consequently, only variants that were inserted by our read simulator were considered as true positives.

**Figure 5:**
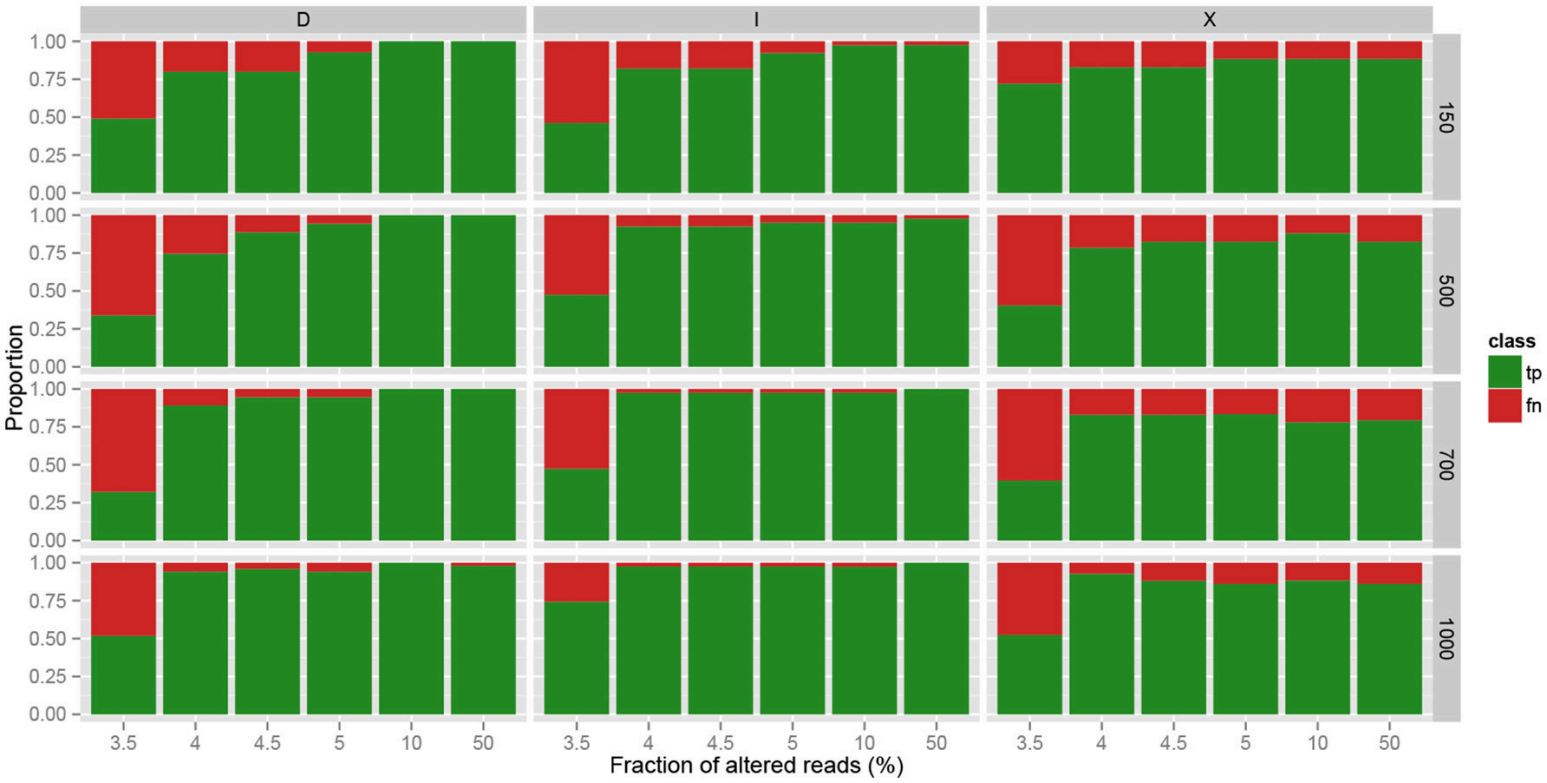
**Accuracy of MICADo calling results on synthetic data**. Proportion of correctly discovered alterations (y-axis) function of the fraction of altered reads (x-axis). Vertical panels report substitutions (“X”), deletions (“D”) and insertions (“I”). Horizontal panels report results for various sample sizes (150, 500, 700, and 1000 reads). We see that MICADo identifies indels with high precision.

We then evaluated in more details the ability of MICADo to identify single nucleotide variants and small indels (<5 nt). We report in Figure 5 calling results for each of the three possible classes of variants (substitutions denoted by “X,” deletions by “D,” and insertions by “I”). We observe that MICADo adequately detects most deletions and insertions, task considered to be difficult for variant calling.

### 3.2. *TP53* targeted data

**Figure 6:**
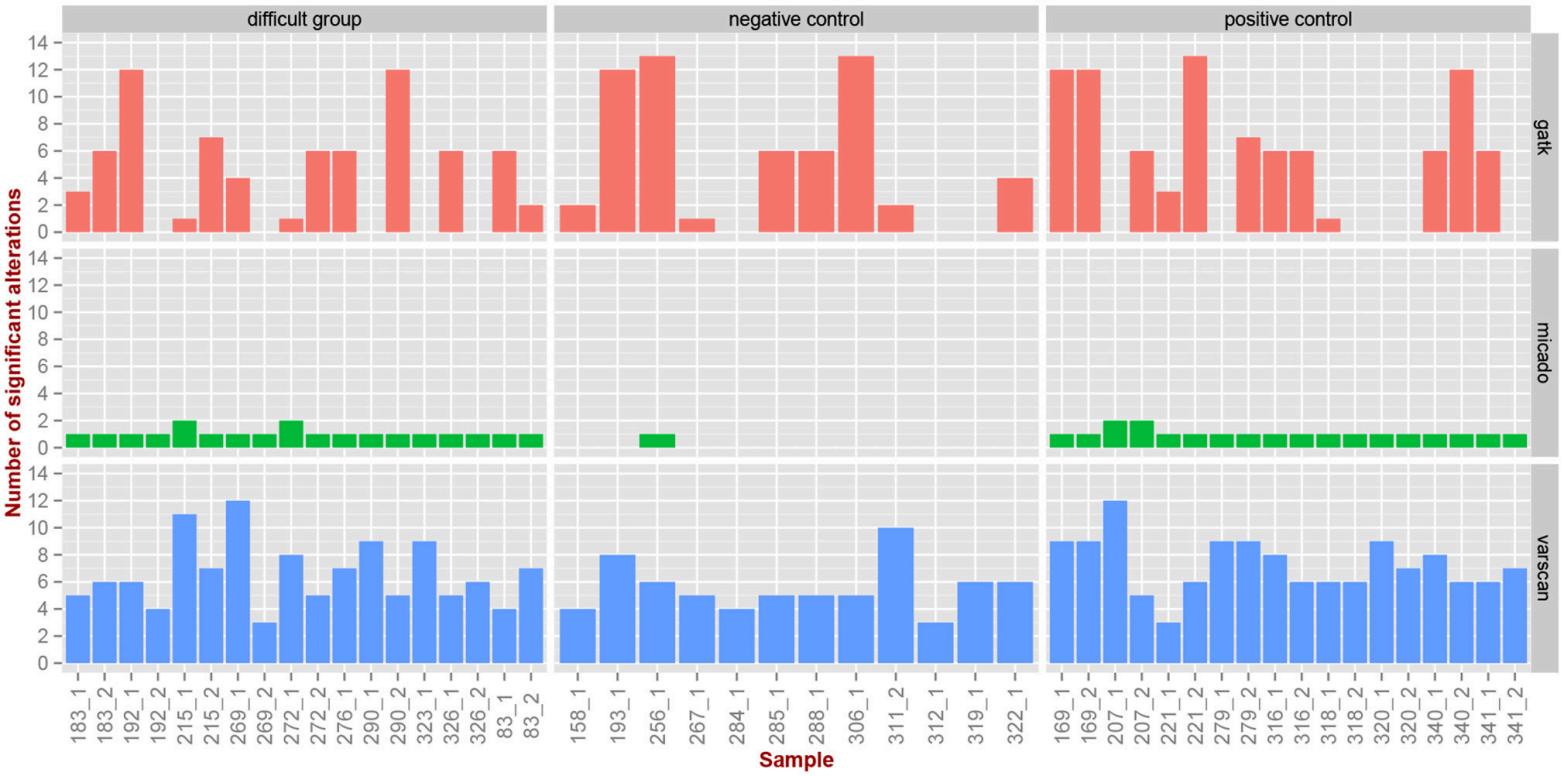
**Comparison of caller's results for TP53 samples**. Three pipelines (vertical panels) were applied on the *TP53* samples (x-axis) to identify alterations in the three groups (horizontal panels). We report here the number of identified significant alterations (y-axis) per category (vertical panels) and per sample.

The three pipelines based on MICADo, VarScan, and GATK, were used to characterize the mutations of the newly sequenced 48 *TP53* samples. See Section 2.2 for details on the samples and Supplementary Section 3 for how these pipelines were run. The results are depicted in Figure 6 and details of the identified alterations are reported in Supplementary Table 1. Note that known polymorphisms affecting the sequencing region were filtered out, either by incorporating known SNPs in the reference graph for MICADo; or by post-processing calls from GATK and VarScan.

We previously reported (Iggo et al., 2013) by independent 454 sequencing that for samples of the difficult group and positive control, we expect one or two significant alterations (mutations) per sample, while we expect no mutations for the negative control group. The only sample that carries two mutations is 276_1, belonging to the difficult group, where MICADo correctly call one of them (the other one is a false negative).

We observe in Figure 6 and Supplementary Table 1 that both GATK and VarScan exhibit high false positive rate, and that the number of identified alterations seems to be independent of the difficulty category. On the contrary, we observe that MICADo adequately limits the false positive rate and that only one sample from the negative control category is identified as being mutated. Indeed, the sample 256_1 exhibits a significant alteration while being in the negative control group. This sample is known to contain a missense variant of doubtful functional significance (Iggo et al., 2013).

Closers inspection (see Supplementary Table 1) shows that alterations identified as significant for 83_1 and 83_2 are in fact false positives (these samples are mutated, but not at the positions identified by MICADo).

We then determined if alterations identified by GATK and VarScan were clustered around hotspots. In our case, we expect loss of function mutations to be uniformly distributed over the transcript. Therefore, if we observe hotspots, these hotspots would likely be due to sequencing errors rather than to alteration recurrence. We thus aggregated the results of the whole cohort and determined wether some alterations were more frequent than others. We detail in Table 1 the top 25 most frequently altered locations, as well as the nucleotidic context before and after the putative alteration. Note that MICADo is absent from this table as by definition it reports no recurrent alterations. We immediately observe in this table that alterations identified by VarScan and GATK are (i) recurrent, with as much as 34 independent samples exhibiting the same deletion, (ii) frequently found in the context of a homopolymer, and (iii) that the deleted nucleotide is one of the homopolymer's nucleotides. This confirms that the sequencing exhibits a higher rate of error in homo-polymeric regions, and that these errors are recurrent across samples.

**Table 1:** **Top 25 “alteration hotspots” along the DNA Binding Domain of TP53 for VarScan (top panel) and GATK (bottom panel)**.

**Pos**	**Before**	**R**	**A**	**Type**	**After**	**Count**
412	AGGCTGCT	TC	T	D	CCCCCCGT	34
1099	ACGAGCTG	GC	G	D	CCCCCAGG	34
652	ATTCCACA	AC	A	D	CCCCCGCC	29
464	TGCACCAG	GC	G	D	CCCCCTCC	28
581	CACGTACT	TC	T	D	CCCCTGCC	16
729	AGGCGCTG	GC	G	D	CCCCCACC	16
1078	GCAAGAAA	AG	A	D	GGGGAGCC	13
767	TGGTCTGG	GC	G	D	CCCCTCCT	12
1147	GCTCCTCT	TC	T	D	CCCCAGCC	11
1201	AGATCCGT	TG	T	D	GGGCGTGA	11
475	CCTCCTGG	GC	G	D	CCCCTGTC	6
652	ATTCCACA	A	AC	I	CCCCCGCC	6
729	AGGCGCTG	G	GC	I	CCCCCACC	6
502	CTTCCCAG	GA	G	D	AAAACCTA	5
1126	GAGCACTG	GC	G	D	CCCAACAA	5
996	AATCTACT	TG	T	D	GGGACGGA	4
452	ACCGGCGG	GC	G	D	CCCCTGCA	26
652	ATTCCACA	AC	A	D	CCCCCGCC	13
412	AGGCTGCT	TC	T	D	CCCCCCGT	11
422	CCCCGTGG	GC	G	D	CCCCTGCA	10
729	AGGCGCTG	GC	G	D	CCCCCACC	10
1099	ACGAGCTG	GC	G	D	CCCCCAGG	9
767	TGGTCTGG	GC	G	D	CCCCTCCT	7
464	TGCACCAG	GC	G	D	CCCCCTCC	5

For each base (column Pos, reference transcript's coordinate), we report in the column Count the number of significant alterations reported by each caller, as well as the context Before, the context After, the Reference sequence and the Alternative sequence. Column Type reports the alteration type: insertions (I) and deletions (D).

Note that both VarScan and GATK were run with stringent minimal base quality threshold (cf. Supplementary Section 3 for details), and that we observed that quality for bases in these homo-polymers very frequently received the highest possible quality score output by PacBio sequencers. This implies that callers relying on quality score to filter out false positives would not be able to avoid these errors.

In summary, these results demonstrate that several alterations that are significant at the individual level (such as those reported by GATK and VarScan) are actually resulting from systematic sequencing biases. With the MICADo algorithm, we measure the specificity of each alteration by contrasting it with background samples generated by resampling, thus accounting for the systematic biases.

### 3.3. *FLT3* targeted data

To further test MICADo, we applied it to the publicly available PacBio sequencing targeted on the *FLT3* kinase domain (see Supplementary Section 4). For each patient, we check for absence of mutations in samples before treatment and we search for at least one mutation in samples after relapse in accordance with Smith et al. (2012). Each sample before treatment and after relapse carries an ITD that are co-localized between each samples but specific to each patient. Normal samples (Normal Control no. 1, no. 2, and no. 3) are not supposed to carry any alterations which was confirmed by MICADo (data not shown). Alteration results obtained by MICADo on the remaining samples were compared with those reported in the original paper, results are shown in Table 2.

**Table 2:** **Results of MICADo variant calls for FLT3 PacBio sequencing data**.

**Subject #**	**Exp. pr #**	**MICADo pr**.	**Exp. pr #**	**MICADo rel**.
1009-003	0	0/0	1	0/0
1011-006	0	0/0	1	1/1
1011-007	0	0/0	2	1/1
1005-004	0	0/1	1	1/2
1005-006	0	0/0	1	1/1
1005-007	0	0/0	1	1/1
1005-009	0	0/0	1	1/1
1005-010	0	0/0	1	1/1

For each sample we provide its expected number of mutations for pretreatment (Exp. pr. #) and relapse states (Exp. rel. #) and the number of correct calls (True Positives) (c) over total calls (t) for pretreatment and relapse states obtained by MICADo (MICADo pr. and MICADo rel., respectively) c/t.

In the original paper, authors have manually analyzed a small region restricted to only 4 codons downstream from the ITD region. We have processed the entirety of the targeted sequences, but focused our analysis on the region after the ITD in the same way as in the original paper. The pre-treatment samples all had the percent of altered reads below 0.43%. We have consequently set the number of expected alterations to be 0 (exp. # column in Table 2). For the relapse samples we have counted only 1 alteration for each altered position, thus taking into the account only the majority clone in our counting of expected alterations.

As can be seen in Table 2 MICADo identified mutations with high precision. Indeed, there are 2 mutation calls that were not previously reported (identified as false positives in the Table 2) and 1 false negative. The second mutation in the subject 1011-007 (relapse) is identified by MICADo, but is filtered out by the *z*-score threshold. The potential false positives in pretreatment and relapse subjects 1005-004 are identical and correspond to a mismatch in a region that has not been analyzed in the original paper. For subjects 1009-003 (pretreatment and relapse), 1011-007 (pretreatment and relapse), and 1005-007 MICADo identified large insertions corresponding to the ITD regions with *k* = 30. In other subjects the duplication can be identified, given *k* > 30.

## 4. Discussion

We describe here a novel method for calling mutations in sequencing data produced for a cohort of patients that makes it possible to distinguish between real mutations and other alterations, including sequencing errors. The originality of MICADo is three-fold. First, it is based on de Bruijn graphs and does not require reads to be aligned on a reference sequence, a step known to be particularly error-prone in the case of indels. Second, MICADo uses cohort information and an exact test to correct for systematic and recurrent alterations caused by technical biases. This has the advantage of allowing correct identification of loss-of-function mutations, which are known to be non-recurrent at the nucleotide level over a cohort, but recurrent in a given sample. Third, by construction, MICADo allows known alterations and multiple reference sequences to be jointly analyzed in a single run.

Our method is especially relevant in the case of clinical targeted PacBio sequencing data for which a generic algorithmic solution has been lacking. MICADo was able to achieve significant improvement over widely used available methods (GATK and VarScan) by controlling both the low false positive and false negative rates.

Our analysis also suggests that PacBio targeted sequencing harbors recurrent errors in homopolymer regions – observation that goes counter to the often admitted hypothesis of uniform distribution of sequencing errors in PacBio reads.

We believe that our method can be a useful addition to the presently available mutation calling tools and could be effectively used in targeted sequencing with high background noise from cohorts of patients.

## Author contributions

RI, JB, and HB conceived the biological study; methods and bioinformatics experimental setup were designed by JR, HS, RU, and MN; algorithms were implemented by JR and HS. Manuscript was written by JR, HS, RU, and MN. All of the authors approved the final manuscript.

## Funding

This work was supported in part by the SIRIC BRIO. JB's research group was supported by grants from the Swedish Cancer Society, Knut and Alice Wallenberg's fund, the research funds at Radiumhemmet, Karolinska Institutet and Stockholm County Council, BRECT and ALF.

### Conflict of interest statement

The authors declare that the research was conducted in the absence of any commercial or financial relationships that could be construed as a potential conflict of interest.
