# Supplementary material for: MICADo – Looking for Mutations in Targeted PacBio Cancer Data: An Alignment-Free Method
Source: Front Genet. 2016 Dec 8;7:214. doi: 10.3389/fgene.2016.00214 (PMC5143680; doi:10.3389/fgene.2016.00214)
Supplement: Supplementary file 1 [file Presentation1.PDF]

Supplementary Material:

MICADo - Looking for mutations in targeted PacBio cancer data: an alignment-free method

Justine Rudewicz [1,2,3*], Hayssam Soueidan [1,2], Raluca Uricaru [1,2], Hervé Bonnefoi [3], Richard Iggo [3], Jonas Bergh [4] and Macha Nikolski [1,2*]

*Correspondence:
Justine Rudewicz
justinerudewicz@gmail.com

1 MICADO IMPLEMENTATION

MICADo is implemented as a python program. This ensures multiplatform compatibility as well as provides access to a number of efficients and widely tested librairies, in particular for bioinformatics applications. MICADo takes as input a reference sequence file, a sample sequence file, an optional file with known SNPs and a path to the directory where all the sequence files for the cohort are stored. On the first execution, all reads from the cohort are pooled together and serialized in a binary format to speed-up the resampling performed during the permutation test. De Bruijn graphs are represented and analyzed using the NetworkX library Schult and Swart (2008).

Notice, that for the construction of de Bruijn graphs the k-mer length k is automatically adjusted so that there is no cycle left in the reference graph. k can be also automatically adjusted in order to avoid cycles in the sample graph or set to the same value as in the reference graph (using the flag `--disable_cycle_breaking`).

The figure S1 shows the principal steps implemented throught the MICADo source code.

2 DATA PREPROCESSING

2.1 TP53 dataset

Reference data

Reference TP53 sequence corresponding to the prototypic p53 isoform NM_000546.5 was used in this study.

To this reference we added the information on known SNPs located in the targeted regions, namely rs1042522, rs137852793, rs137852792, rs121912665 and rs1800372. In order to obtain SNP positions on transcripts we used SNPnexus (Chelala et al., 2009). From these positions we constructed an SNP file using a python script (see `make_SNP_file.py` script in the `bin` directory and `snp_TP53.tab` SNP file on the github repository) in the `data/reference` directory.

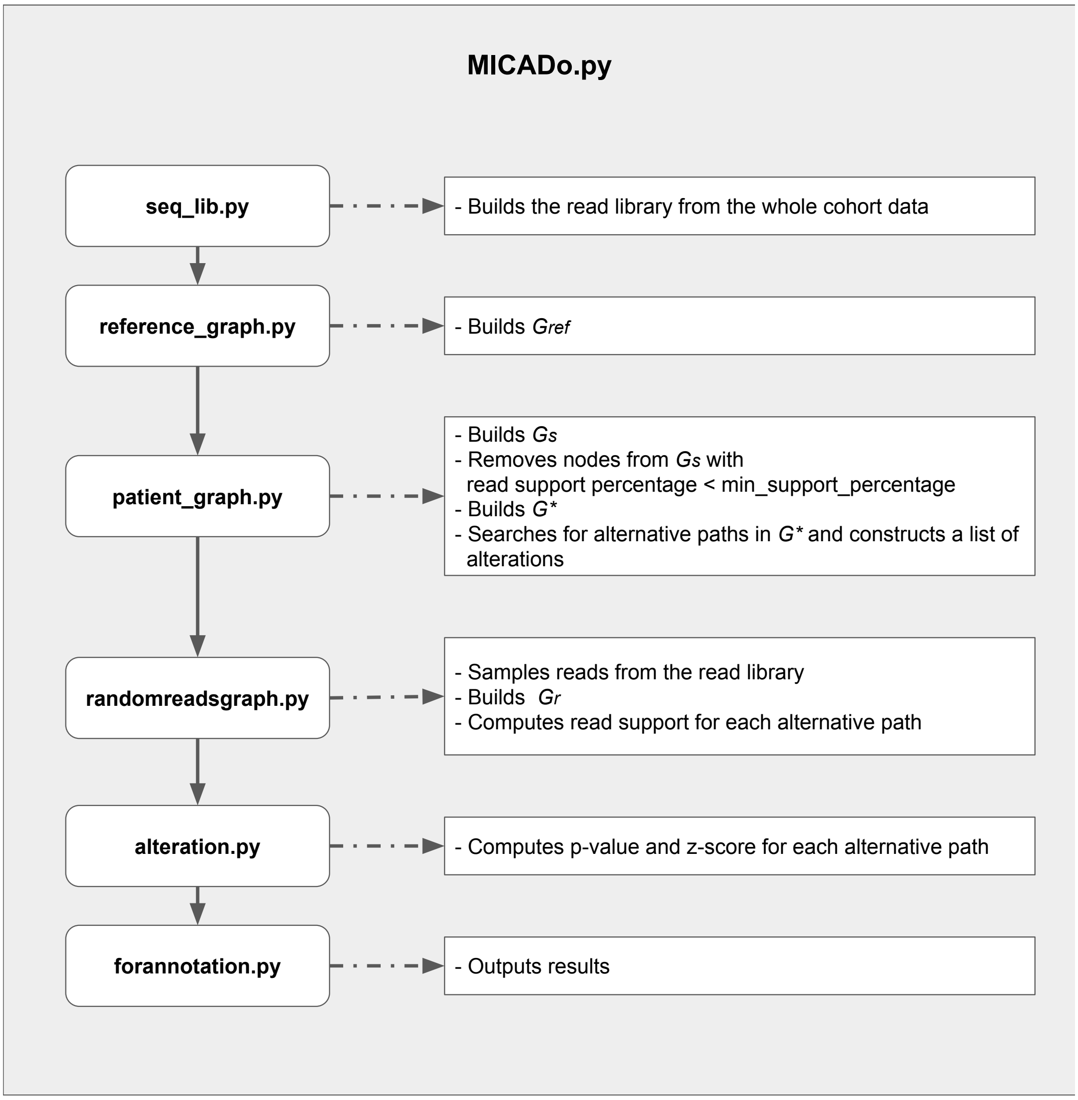

Figure S1. *MICADo workflow.* Description of the main steps of the source code of MICADo.

Sequencing data

TP53 sequencing was performed at the National Genomics Infrastructure (Uppsala University) on 1277 patients of the prospective clinical trial EORTC 10994/BIG 1-00. It was centered on the DNA Binding Domain of p53 protein which was split into two separate fragments for the nucleotides 159-642 and 590-1045 in the TP53 mRNA corresponding to 54-348 TP53 codons on the p53 protein. Data are available separately for each of the two fragments and can be retrieved from SRA under the accession number SRP064161 BioProject PRJNA290142. In the current study we focused on the 48 sample subset that has been previously analyzed using 454 Roche sequencing. Numbers for the 48 samples are given e.g. in Supplementary table S1.

Prior to the analysis we preprocessed the fastq files: we deleted tags used for multiplexing using cutadapt (Martin, 2011) and reversed reads when needed (see the `bin/orient_reads_in_forward_direction.py` program on github).

2.2 FLT3 dataset

FLT3 reference sequence corresponds to its unique isoform: NM_004119 and the 4 SNPs are rs121913491, rs121913232, rs121913487, and rs147467327 (see `reference_FLT3.fasta` file in the `data/reference` directory). As for TP53, we used SNPnexus to learn SNP positions on the transcript and built an SNP file (see the `snp_FLT3.tab` SNP file in the `data/reference` directory on the github).

Prior to the analysis all reads have been oriented to be on the forward strand using the `orient_reads_in_ forward_direction.py` script in the `bin` directory (see the github repository).

3 RUNNING VARSCAN GATK AND MICADO ON TP53 AND SYNTHETIC DATA

As stated in introduction, we tested GATK and VarScan because they are widely used to identify sequence variants. However, we did not expect them to perform well on samples with frequent artefactual indels. In particular, for samples without a matching normal control, VarScan simply lists the sequence variants present above a certain frequency in a pileup of the data. It does not perform sophisticated statistical tests based on the entire cohort, so it is not surprising that it does not filter out obvious recurrent artefacts. Our results show that MICADo performs vastly better than the current implementations of both GATK and VarScan, and justify its continued development and application for PacBio data.

Results of VarScan, GATK and MICADo are compared with mutations previously identified in (Iggo et al., 2013). Information on these mutations is gathered in `data/JPath2013_mutations.tsv` on the github repository.

All commands were organized using the Snakemake engine Köster and Rahmann (2012), and all the workflows are available in the github repository.

VarScan, GATK and the synthetic data generator requires mapped reads. Mapping was done with GMAP (v.2015-09-25) (Wu and Watanabe, 2005) against the TP53 mRNA sequence NM_000546.5 with the parameter `min-intronlength=15000` to prevent intron calling and `-f samse` option to obtain output in the samtools format (see rule `gmap_align_sample` of the `Snakemake_tools` file on github). The alignment tool (that is, GMAP), was choosen after comparing alterations count present in sam alignment files obtained by three major aligners, namely BWA (Li, 2013), BLASR (Chaisson and Tesler, 2012) see figure S2. A perl script (`bin/parsesam.pl`) was used to parse sam files.

We used samtools (v.1.2.0) to convert alignment files in bam format, sort and index them.

VarScan v.2.4.0 (Koboldt et al., 2012) was ran on the mpielup file generated by samtools 1.2.0 with the following parameters : (i) a minimum read coverage of 5, (ii) frequency of alterated nucleotides of 0.05, (iii) minimal quality of 60 and p-value threshold of 0.001. See rule `varscan_call` of the `Snakemake_tools` file on github.

A pipeline based on GATK v.3.4-46-gbc02625 was developed following guidelines for single sample calling DePristo et al. (2011). The pipeline is based on the following steps: (i) `SplitNCigarReads`, (ii) `RealignerTarget Creator`, (iii) `IndelRealigner` and (iv) `HaplotypeCaller` in GVCF mode. Only variants with a `NON_REF` identified alteration were selected for further analysis. See rule `gatk_call` of the `Snakemake_tools` file on github for details on the command use.

MICADo was run on the TP53 samples with the following parameters: (i) k-mer length of 18 (determined as the minimal k-mer length yielding DBG without cycles in the reference), (ii) 1000 permutations and (iii)

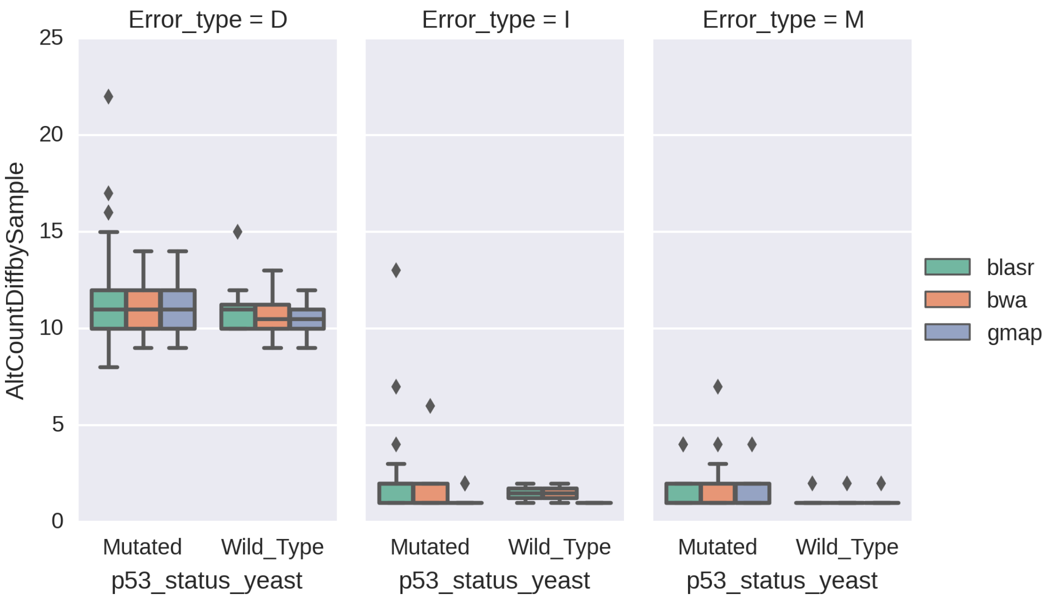

Figure S2. *Alterations count by sample.* Number of different alteration by sample. Alterations are categorized by error type, insertion (I), deletion (D) and mismatch (M) depending on p53 statut. Only alterations with a read support > 0.05 and quality > 40 are shown.

a minimal p-value of 0.01. See rule `micado_call` of the `Snakemake_tools` file on github for details on the command use. MICADo outputs a `json` file describing the parameters used for execution, the list of identified alterations as well as an annotated list of significant alterations (those below the p-value threshold parameter and a minimal z-score of 10).

For the synthetic dataset, we compared the results of the three callers against the known position and type of alterations generated by the sampler using the `tabulate_and_aggregate_xp_results.py` program in `bin` folder.

All figures resulting from our evaluations are generated using `R` scripts provided in the github repository (`manuscript/analysis` folder).

The figure S3 shows the execution time comparison for the GATK, MICADo and VarScan pipelines as a function of the number of reads. Execution time of MICADo appears reasonable although VarScan shows the best execution time.

4 RUNNING MICADO ON FLT3 DATASET

MICADo was run on the FLT3 samples with the following parameters: (i) k-mer length of 30, (ii) 1000 permutations and (iii) the parameter `--disable_cycle_breaking` to discard cycles (see `Snakefile_flt3` file on github for details on the command use). Indeed, consistently with the original paper, we looked for mutations outside of the ITD mutations region (see `data/Nat2012_mutations.tsv` on the github repository.). We have used minimal p-value of 0.001 and a minimal z-score of 5.

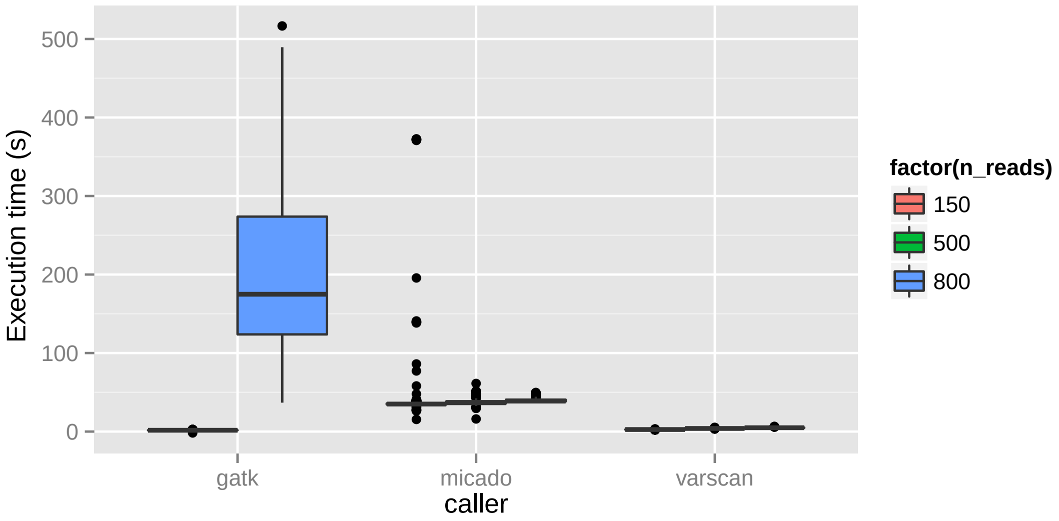

Figure S3. *Execution time of the three variant callers.* The execution time of GATK, MICADo and VarScan are shown as a function of the number of reads.

5 REPRODUCIBILITY

All results presented in this paper can be reproduced using the git version tagged 'v.1.0'.

6 SYNTHETIC DATASET GENERATION

Synthetic data were generated using the `altered_reads_sampler.py` python program. This program receives as input a SAM file consisting of all the reads from the negative control group that were mapped on the TP53 reference transcript using GMAP. To generate synthetic random samples, we ran the sampler program with the following parameters: (i) max indel length was set to 5, (ii) number of reads ranges between 150 and 700, (iii) number of alterations to inject ranges from 1 to 3, (iv) fraction of altered reads ranges between 3.5% to 50%. All parameters are set in the `generate_synthetic_reads` Snakemake rule from the `Snakefile_synth` file on github.

The read sampler outputs two files, (i) a `fastq` file containing both non-altered and altered reads (depending on the fraction altered parameter) as well as a `json` file summarizing the sampling and indicating the exact location and nature of the alterations.

7 SUPPLEMENTARY RESULTS FOR TP53 DATA

We can see on figure S4 that the size of the de Bruijn graph representation of a sample – measured as the size of the vertex set $|V_s|$ – is correlated with read abundance before the removal of k-mers with read support below a fixed threshold (depicted in blue). However, after removal of such vertices this correlation disappears (depicted in red). Basically, this cleaning step removes completely random sequencing errors and thus the size reduction is function of noise present in the sample's reads.

	EORTC ID	category	Exp. #	MICADo	GATK	VarScan
1	158_1	negative control	0	0/0	0/1	0/4
2	193_1	negative control	0	0/0	0/3	0/8
3	256_1	negative control	1	1/1	1/4	1/6
4	267_1	negative control	0	0/0	0/1	0/5
5	284_1	negative control	0	0/0	0/0	0/4
6	285_1	negative control	0	0/0	0/2	0/5
7	288_1	negative control	0	0/0	0/2	0/5
8	306_1	negative control	0	0/0	0/4	0/5
9	311_2	negative control	0	0/0	0/1	0/10
10	312_1	negative control	0	0/0	0/0	0/3
11	319_1	negative control	0	0/0	0/4	0/6
12	322_1	negative control	0	0/0	0/2	0/6
13	169_1	positive control	1	1/1	1/3	1/9
14	169_2	positive control	1	1/1	1/3	1/9
15	207_1	positive control	1	1/2	1/5	1/12
16	207_2	positive control	1	1/2	1/2	1/5
17	221_1	positive control	1	1/1	1/2	1/3
18	221_2	positive control	1	1/1	1/4	1/6
19	279_1	positive control	1	1/1	1/5	1/9
20	279_2	positive control	1	1/1	1/3	1/9
21	316_1	positive control	1	1/1	1/2	1/8
22	316_2	positive control	1	1/1	1/2	1/5
23	318_1	positive control	1	1/1	1/1	1/6
24	318_2	positive control	1	1/1	1/5	1/6
25	320_1	positive control	1	1/1	1/5	1/9
26	320_2	positive control	1	1/1	1/4	1/7
27	340_1	positive control	1	1/1	1/2	1/8
28	340_2	positive control	1	1/1	1/3	1/6
29	341_1	positive control	1	1/1	1/3	1/6
30	341_2	positive control	1	1/1	1/6	1/7
31	183_1	difficult group	1	1/1	0/2	1/5
32	183_2	difficult group	1	1/1	0/2	1/6
33	192_1	difficult group	1	1/1	1/3	1/6
34	192_2	difficult group	1	1/1	1/4	1/4
35	215_1	difficult group	1	1/2	1/1	1/11
36	215_2	difficult group	1	1/1	1/3	1/7
37	269_1	difficult group	1	1/1	1/2	1/12
38	269_2	difficult group	1	1/1	1/6	1/3
39	272_1	difficult group	1	1/2	1/1	1/8
40	272_2	difficult group	1	1/1	1/3	1/5
41	276_1	difficult group	2	1/2	1/2	1/7
42	290_1	difficult group	1	1/1	0/4	1/9
43	290_2	difficult group	1	1/1	0/3	1/5
44	323_1	difficult group	1	1/1	0/0	0/9
45	326_1	difficult group	1	1/1	1/2	1/5
46	326_2	difficult group	1	1/1	1/4	1/6
47	83_1	difficult group	1	0/1	0/2	0/3
48	83_2	difficult group	1	0/1	0/1	0/7

Table S1 *Results of MICADo variant calls for TP53 PacBio sequencing data.* For each sample we provide information on its *category* in the yeast assay test, the expected number of mutations in a sample (exp.#), and the number of correct calls (True Positives) (c) over total calls (t) for each of tested pipelines, reported as fraction c/t.

REFERENCES

Chaisson, M. J. and Tesler, G. (2012). Mapping single molecule sequencing reads using basic local alignment with successive refinement (blasr): application and theory. *BMC bioinformatics* 13, 238

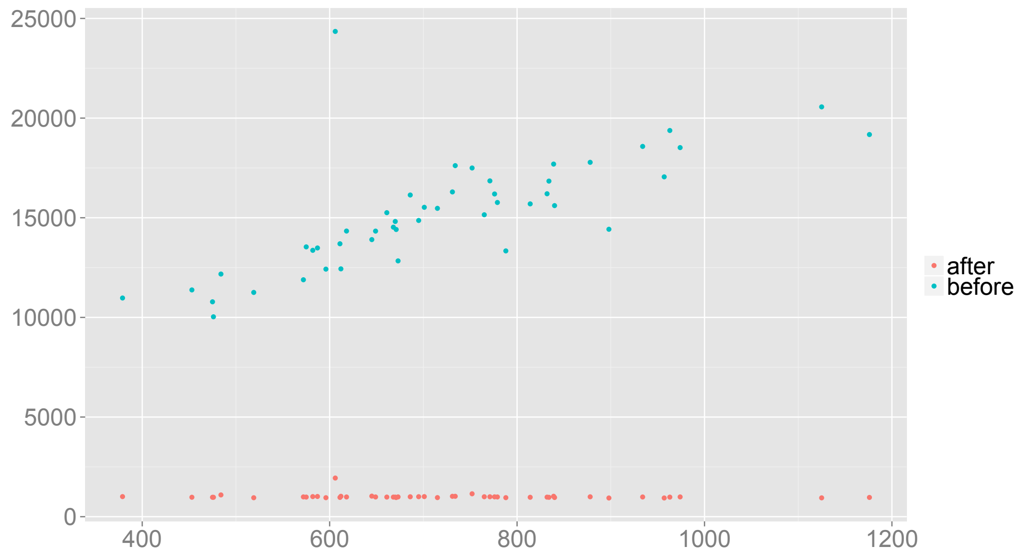

Figure S4. *Size of de Bruijn graphs per sample.* Number of different k-mers for $k = 16$ per sample (y-axis) function of number of sequencing reads (x-axis) for the TP53 dataset. Data are represented before (blue dots) and after (red dots) removing k-mers whose read support is under the threshold t fixed to 3%.

Chelala, C., Khan, A., and Lemoine, N. R. (2009). Snpnexus: a web database for functional annotation of newly discovered and public domain single nucleotide polymorphisms. *Bioinformatics* 25, 655–661

DePristo, M. A., Banks, E., Poplin, R., Garimella, K. V., Maguire, J. R., Hartl, C., et al. (2011). A framework for variation discovery and genotyping using next-generation dna sequencing data. *Nature genetics* 43, 491–498

Iggo, R., Rudewicz, J., Monceau, E., Sevenet, N., Bergh, J., Sjoblom, T., et al. (2013). Validation of a yeast functional assay for p53 mutations using clonal sequencing. *The Journal of pathology* 231, 441–448

Koboldt, D. C., Zhang, Q., Larson, D. E., Shen, D., McLellan, M. D., Lin, L., et al. (2012). Varscan 2: somatic mutation and copy number alteration discovery in cancer by exome sequencing. *Genome research* 22, 568–576

Köster, J. and Rahmann, S. (2012). Snakemakea scalable bioinformatics workflow engine. *Bioinformatics* 28, 2520–2522

Li, H. (2013). Aligning sequence reads, clone sequences and assembly contigs with bwa-mem. *arXiv preprint arXiv:1303.3997*

Martin, M. (2011). Cutadapt removes adapter sequences from high-throughput sequencing reads. *EMBnet. journal* 17, pp–10

Schult, D. A. and Swart, P. (2008). Exploring network structure, dynamics, and function using networkx. In *Proceedings of the 7th Python in Science Conferences (SciPy 2008)*. vol. 2008, 11–16

Wu, T. D. and Watanabe, C. K. (2005). Gmap: a genomic mapping and alignment program for mrna and est sequences. *Bioinformatics* 21, 1859–1875
